# Urea production via photocatalytic coupling of mixed gases (CO_2_/NH_3_) using Mo(MnO_4_)_5_ supported on Ce-BTC as nano-composite catalyst

**DOI:** 10.1038/s41598-024-65363-z

**Published:** 2024-07-06

**Authors:** Mahmoud El-Shahat, Reda M. Abdelhameed

**Affiliations:** 1 Photochemistry Department, Chemical Industries Research Institute, National Research Centre, 33 El Buhouth St., Dokki, Giza, 12622 Egypt; 2 Applied Organic Chemistry Department, Chemical Industries Research Institute, National Research Centre, 33 El Buhouth St., Dokki, Giza, 12622 Egypt

**Keywords:** Metal organic framework, Cerium, Photocatalysis, Urea, Ammonia, Carbon dioxide, Chemistry, Catalysis, Heterogeneous catalysis

## Abstract

Urea used in fertilization and feed supplement, as well as a starting material for the manufacture of plastics and drugs. Urea is most commonly produced by reacting carbon dioxide with ammonia at high temperature. Photocatalysis has gained attention as a sustainable pathway for performing urea. This work focus on designing very active photocatalysts based on cerium organic framework (Ce-BTC) doped with metal oxide nanoparticles (molybdenum permanganate, Mo(MnO_4_)_5_) for production of urea from coupling of ammonia with carbon dioxide. The prepared materials were characterized using different spectral analysis and the morphology was analysed using microscopic data. The effect of catalyst loading on the production rate of urea was investigated and the obtained results showed speed rate of urea production with high production yield at low temperature. The recyclability tests confirmed the sustainability of the prepared photocatlysts (Mo(MnO_4_)_5_@Ce-BTC) which supported the beneficial of the photocatalysis process in urea production.

## Introduction

Urea was one of the commercially available sources of nitrogen fertilizers^1–3^. It is not only broadly applied in agriculture but also in industrial production as an indispensable raw material ^4^ in synthesis of herbicides or pesticides ^5^, plastics, as a fire retardant, in tobacco goods, some wines, and the cosmetics industry^6^. Urea was added to fuels, and it cooperates with SCR systems to reduce NOx emissions from combustion engines ^7,8^. Moreover, it was used as intermediate in a wide range of agrochemicals, dyes, and resin precursors ^9^. Urea based-pharmaceutical compounds showed antibacterial ^10^, antiatherosclerotic ^11^, antidepressant ^12^, and anticancer ^13–16^.

Recently, alternative routes for direct urea production have been developed using catalysis pathways^17–21^. The oxidative carbonylation of alkylamines using PdI_2_ as catalyst in the presence of O_2_, which afforded the corresponding urea in good yields ^22^. Palladium nanoparticles loaded on molecular sieve LTA-3A, denoted as Pd/LTA-3A. The Pd nanoparticles catalyze the adsorption of NH_3_ to Pd-NH_2_ and Pd-H surface species, which react with CO_2_ and NH_3_ to form urea^23^. Acyclic and cyclic ureas from aromatic primary amines, using *N,N′-*bis(salicylidene)ethylenediaminocobalt(II) [Co(salen)] as catalyst ^24^. Polymer-immobilized gold catalysts were found to catalyze the carbonylation of arylamines with CO_2_ to their diarylureas in the absence of organic solvents^25^. W(CO)_6_-catalyzed oxidative carbonylation of diamines to cyclic ureas ^26^. Catalytic approach prevents the use of lethal carbon monoxide directly and allows the production of simple and functionalized urea derivatives with hydrogen liberation from amines using ruthenium-catalyzed N–H activation, carbon monoxide insertion from DMF, and dihydrogen liberation^27^. Utilising a pincer-supported iron catalyst, a series of urea was generated with isolated yields of up to 80% and the only byproduct being H_2_^28^. Direct and selective urea production from nitrate and carbon dioxide under normal condition using a more ecologically friendly electrocatalytic process that uses an indium hydroxide catalyst^29^. One-pot green urea electrosynthesis can be used to make urea instead of the traditional method. To significantly improve urea synthesis from CO_2_ and NO_3_^-^ utilising the co-reductive C-N coupling strategy in natural settings, a bifunctional indium hydroxide ln–O–x–O–ln (x = C or N) was developed^30^. The majority of the preparation of urea uses the CO_2_ and ammonia as reagents. This traditional process requires complex and difficult reaction conditions^31^. Therefore, the need for a straightforward urea synthesis process under safe conditions arises. As a quick, simple, and environmentally friendly process, photocatalytic synthesis technology has gained popularity recently^32,33^. Titanium dioxide nanocrystals (Q-TiO_2_) immobilised in a layer of polyvinylpyrrolidone gel (Q-TiO_2_/PVPD) were utilised in the first photocatalytic report of urea synthesis to reduce CO_2_ and nitrate ion (NO_3_^-^)^34^. A film of TiO_2_ embedded in SiO_2_ matrices (Q-TiO_2_/SiO_2_) proposed the photochemical reduction of CO_2_ saturated in a LiNO_3_ solution generated urea, using 2-propanol as an electron donor and the system was irradiated for 5 h with a 500W mercury lamp ^35^.Incoporating copper (Cu) nanodeposits on TiO_2_ particles, using CO_2_ and NaNO_3_ as a precursor for photocatalyzed urea synthesis, where polyvinyl alcohol fulfills the role of electron donor and followed by 5 h of irradiation with a 200WXg-Helamp ^36^. A single Cu atom anchored to TiO_2_ catalysed the photoelectrocatalytic urea production using CO_2_ and nitrate depend on the short distances between a dual active metal sites Cu and Ti^37^. It has been discovered that TiO_2_-immobilized reversible single-atom copper (abbreviated as Cu SA-TiO_2_) can be used to accelerate electron-transfer dynamics and boost the efficiency of urea's photosynthesis from N_2_, CO_2_, and H_2_O^38^. The effect of the different ratio of poly(styrene sulfonate)/poly(allylamine hydrochloride) capsules of different size on the TiO_2_-assisted photosynthesis of urea from inorganic precursors (CO_2_ and NO_3_^-^) in aqueous solution was demonstrated, where, Poly(vinyl alcohol) was employed as electron donor to facilitate the photosynthetic process^39^. TiO_2_ stabilized in perfluorodecalin (PFD:TiO_2_) emulsions and 2-propanol as electron donor species, A 120 W mercury vapour lamp was utilized as a light source, the concentration of urea was 0.54 mM after one hour of irradiation with^40^. TiO_2_ and Fe-titanate based on zeolite (Fe-titanate HZSM-5) were synthesized and used in the batch reactor under UV irradiation to create urea through photocatalytic reduction of nitrate in water and isopropanol/oxalic acid as hole scavengers. When using 1% isopropanol-containing solution over 10% Fe-titanate HZSM-5 photocatalyst, the maximum output of urea was 18 ppm^41^. For the synthesis of urea, carbon nanotubes (CNTs) with Fe/Ti^3+^-TiO_2_ composite were used as the photocatalyst (Ti^3+^-TiO_2_ /Fe-CNTs). The photocatalytic synthesis of urea in water from the reduction of N_2_ and CO_2_ was accomplished using these generated catalysts (Ti^3+^-TiO_2_ /Fe-CNTs). Additionally, using a 300W high-pressure mercury lamp as the light source, the urea yield can reach 710.1 mol/(L g) in a 4 h reaction^19^. The urea conversion rate from N_2_, CO_2_, and H_2_O using NiCoP (NCP) nanoparticles as a co-catalyst and flower-shaped ZnIn_2_S_4_ microspheres as a photocatalyst was 13.9 mol g/h (15%-NCP/ZIS), but it increased to 19.6 mol g/h (15%-NCP/ZIS) with mildly rising pressure (10 bar) and temperature (80 °C)^42^.

Recently, a porous crystalline material with good thermal stability known as Metal–Organic Frameworks (MOFs) was created by bridging the coordination of metal and organic groups while adding inorganic nodes^43–46^. Due to their regulated morphology and stable structure^47–56^, MOFs have attracted particular attention from researchers for their effectiveness in photocatalysis^57–69^; Ce-BTC is one of the most efficient catalysts^70^. Cerium, on the other hand, has two main oxidation states because it is a member of the lanthanide family. Many different catalyst designs often use the strong oxidant diamagnetic Ce(IV)^71,72^, It effectively interacts with numerous chemical molecules to form Ce(III) compounds ^73,74^. In order to create materials with magnetic ordering ^75^ or scintillation qualities ^76^, paramagnetic Ce(III) gives rise to compounds with characteristic magnetic^77,78^ and luminescent abilities^79,80^. Ce-based MOF (MOF-589) showed higher catalytic efficiency for methylene blue degradation ^81^. Multivalent Ce-MOFs (Ce-UiO-66 and Ce-MOF-808) have shown increased catalytic efficiency, better stability, and recyclability in the oxidation of phenolic compounds when utilised as biomimetic laccase nanozymes for environmental remediation^82^. Ultrasound was used to help produce a CoFe_2_O_4_/Ce-UiO-66 nanocomposite for the photocatalytic aerobic oxidation of aliphatic alcohol^83^. Zirconium-cerium-MOFs with amino-functionalized linkers have been created as effective non-noble-metal-based heterogeneous catalysts for both the aerobic photooxidation of benzylic alcohols and the microwave activation *N*-alkylation of amines with alcohols ^84^. MOF-808(Zr/Ce) was developed specifically for phosphate detection and elimination ^85^. In MOF-808(Ce), dibenzothiophene completed oxidative desulfurization via coordination formic acid^86^. Ce^III^BTC and Ce^IV^BTC, were designed for superoxide radical (O_2_^•–^) elimination and ionizing radiation protection ^87^. Ce(IV)-MOF had a good Chemiluminescence performance towards SO_3_^2−^^88^. A novel Cataluminescence sensor based on Ce(IV)-MOF was designed for detection of H_2_S^89^. The adsorption performance of cerium-based MOFs (Ce-MOF-66 and Ce-MOF-808) towards arsenic species from water was investigated^90^. Composite films made of porous anodic oxide (PAO) and ce-MOFs improved electrodeposition and corrosion resistance ^91^. Ce-MOF-808 can electrocatalyze the electrochemical pre-oxidation of dopamine (DA) to dopaquinone (DAQ) in buffer solutions containing 3-(N-morpholino)propanesulfonic acid^92^. Metal–organic frameworks (MOFs) based on cerium(IV) have gained more and more attention in both the scientific and commercial communities due to their unique potential in fields like photocatalysis and redox catalysis.Additionally, the photocatalytic activity of these Ce(IV)-MOFs for the decarboxylative oxygenation of 4-fluorophenylacetic acid to produce the corresponding C-O bond-forming products 4-fluorobenzaldehyde and 4-fluorobenzyl alcohol was investigated under the illumination of blue light-emitting diodes (LEDs) and in the presence of air^93^. When exposed to sunlight, UiO-66(Ce)-NDC was found to be a powerful CO_2_ chemical fixation catalyst for total water splitting ^94^.

Production of urea based-photocatalysts is still under achievements. Herein, Ce-BTC, Mo(MnO_4_)_5_, and Mo(MnO_4_)_5_@Ce-BTC were synthesized as heterojunctions photocatalyst. The special properties of the Mo(MnO_4_)_5_@Ce-BTC composites leads to choose it as model photocatalyst to investigate the production of urea.

## Experimental

### Materials and reagents

While each agent was of the analytical grade, no additional purification was necessary. 1,3,5-benzenetricarboxylic acid (H_3_-BTC, C_9_H_6_O_6_, 98%), Molybdenum pentachloride (MoCl_5_), Potassium permanganate (KMnO_4_) , cerium nitrate (Ce(NO_3_)_3_.6H_2_O, 99%), ethanol (CH_4_O, 99.7%), and N,N-dimethylformamide (DMF, C_3_H_7_NO,99.5%) were obtained from Aldrich Sigma.

### Synthesis of Ce-BTC MOF

Ce-BTC MOF: Ce-MOFs was synthesized based on the following procedure, 1.066 g (8 mmol) of Ce(NO_3_)_3_.6H_2_O, 1.328 g (8 mmol) of 1,3,5-benzene tricarboxylic acid were dissolved in 10 mL of DMF, respectively. The mixture was refluxed at 125 °C for 8 h. The product was filtered off, washed with DMF. The raw product will disperse in 400 mL mixture of methanol/water (50/50, v/v). The mixture was heated at 100 °C for 12 h, followed by filtration, 3 washing times and drying.

### Synthesis of Mo(MnO_4_)_5_

Molybdenum pentachloride (MoCl_5_, 10 mmol) in 50 mL EtOH was allowed to react for 24 h with potassium permanganate (KMnO_4_, 10 mmol) in 50 mL distilled water (DW). The formed solid was filtered off, washed with EtOH (3 × 20 mL). The mixture was heated at 100 °C for 12 h, followed by filtration, three washing times and drying to obtain a new synthesized material Mo(MnO_4_)_5_.

### Synthesis of Mo(MnO_4_)_5_@Ce-BTC

Mo(MnO_4_)_5_@Ce-BTC nanocomposites were prepared as follow: 0.50 g Ce-BTC was added to 50 mL of ethanol at 25 °C, the mixture was added in ultrasonic bath (UL TRASONS-H, 10 W, 40 kHz) for 1 h. Mo(MnO_4_)_5_ (0.10 g) was dissolved in 50 mL of methanol at 25 °C then added to the solution of MOFs in the same ultrasonic bath for 1 h. The mixture was taken from ultrasonic bath and stirred at 500 rpm for 24 h at room temperature. The sample was recovered by centrifuge (5000 rpm for 20 min) and washed three times with methanol, and then the product was dried under vacuum at 60 °C for 18 h.

### Characterization

The materials' X-ray diffraction (XRD) patterns were examined using (X'pert pro-Panalytical, Holland), operating system with Cu Ka irradiation at 40 kV and 40 mA. The data were gathered in the 2 θ with a step width of 0.05° and a range of 3° to 80°. Field emission- scanning electron microscopy was used to examine the surface morphology of Ce-BTC, Mo(MnO_4_)_5_, and Mo(MnO_4_)_5_@Ce-BTC (with an accelerating voltage of around 20 kV FE-SEM QUANTA FEG250, Republic of Czech). Using a FE-SEM model (AMETA version) and energy dispersive X-ray (EDAX) analysis, the components that made up the nanocatalyst were identified. High resolution transmission electron microscopy (HR-TEM; JEOL, JEM 2100, 120 kV acceleration voltages, Japan) was used to analyse the particle size. Diffuse reflectance spectra (UV–vis DRS) spectrophotometer (model: JASCO 570) in the wavelength range of 190–2500 nm were used to acquire the optical characteristics. Brunauer–Emmett–Teller (BET) surface areas were investigated using Quantachrome autosorb-iQ-2MP. Prior to BET analysis, the materials were degassed in a vacuum chamber for 3 h at 300 °C. Temperatures between 498 and 298 K were used for N_2_ adsorption, with a total flow rate of 20 mL/min. Using a conventional three-electrode probe in an electrochemical workstation, all electrochemical tests were performed at room temperature. The working electrode was a glassy carbon electrode with a geometric surface area of 1.766 mm^2^. It was well polished, rinsed in deionized water, ultrasonically in 100% ethanol, and left to dry briefly at room temperature.

### Photocatalytic experiments

**Figure 1:**
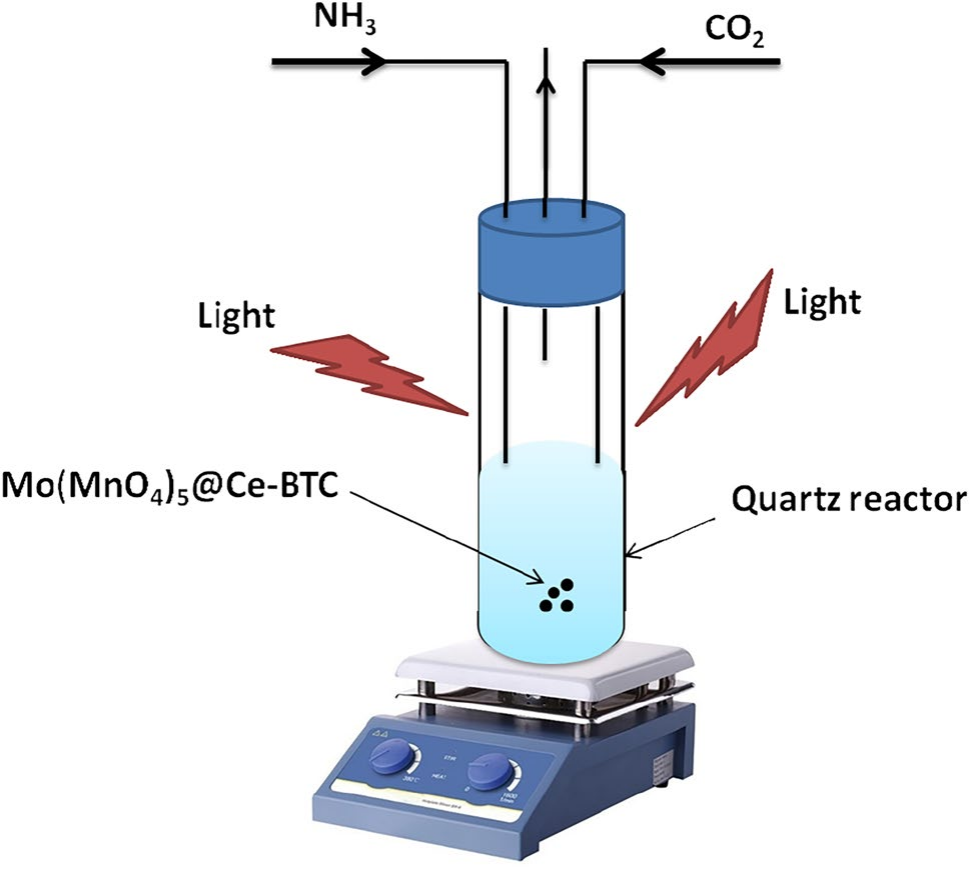
Schematic of the experimental device for photocatalytic urea production.

The catalyst was dispersed into a quartz reaction bottle (double-walled immersion well reactor, where Fig. 1 illustrate the experimental device for photocatalytic urea production), and the reaction system was continuously fueled with NH_3_, and CO_2_. The temperature during the process was controlled using cooling. The photocatalytic synthesis of urea with catalyst was started after stirring for 30 min in the dark; two daylight lamp (300 W, average luminous intensity 1340 lm) as visible light lamp was used. The lamps placed outside the glass immersion well. Every 15 min during the reaction time, a liquid sample was taken, and the supernatant was obtained using centrifugation and membrane filtration.

### The detection process of urea formation

#### Utilising the indophenol blue-urease method to measure the amount of urea in liquid products^95^

Five millilitres (5 mL) of the reaction solution was taken in the two 10 millilitre test tubes, A and B, respectively. After that, 1 mL of the 20 mg/L urease solution was added to tube A and thoroughly shaken, and 1 mL of deionized water was added to tube B and thoroughly shaken as well. The tubes A and B were then placed in the thermotank for 1 h at 50 °C, allowing urease to completely hydrolyze the urea in tube A into NH_4_^+^. The indophenol blue reagent was put into tubes A and B after cooling to room temperature. The absorbance curve of the reaction solution at 500–800 nm was measured and integrated using the spectrophotometer after around one hour. The pertinent parameter of the NH_4_^+^ concentration was the integral area of the absorbance curve. The integrated area of the curve measured by the liquid in the tube A represents the reaction liquid's total ammonia content, while the integrated area of the curve measured by the liquid in the tube B only represents the NH_4_^+^ product. In order to determine the yield of urea, the integral area of the absorbance curve of tube B is subtracted from that of tube A.

#### The standard curve

**Figure 2:**
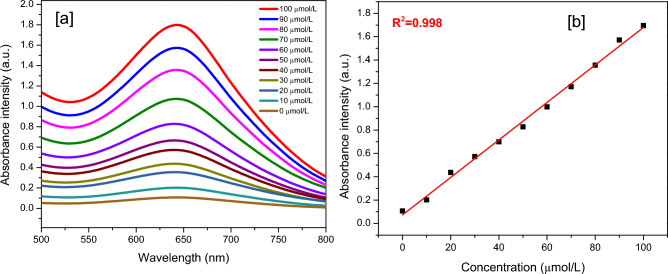
(**a**) The absorbance of NH_4_^+^ with different concentration, (**b**) the standard curve of NH_4_^+^ with different concentration followed the different area of absorption curve.

The NH_4_^+^ standard solution of 0, 10, 20, 30, 40, 50, 60, 70, 80, 90, and 100 μmol/L was coloured via the indophenol blue method, and the absorbance curve is scanning at 500–800 nm as represented in Fig. 2a. The relationship between the concentration of NH_4_^+^ and the integrated area of the absorption curve is shown in Fig. 2b.

## Results and discussion

### Optimizations and characterizations of catalysts

**Figure 3:**
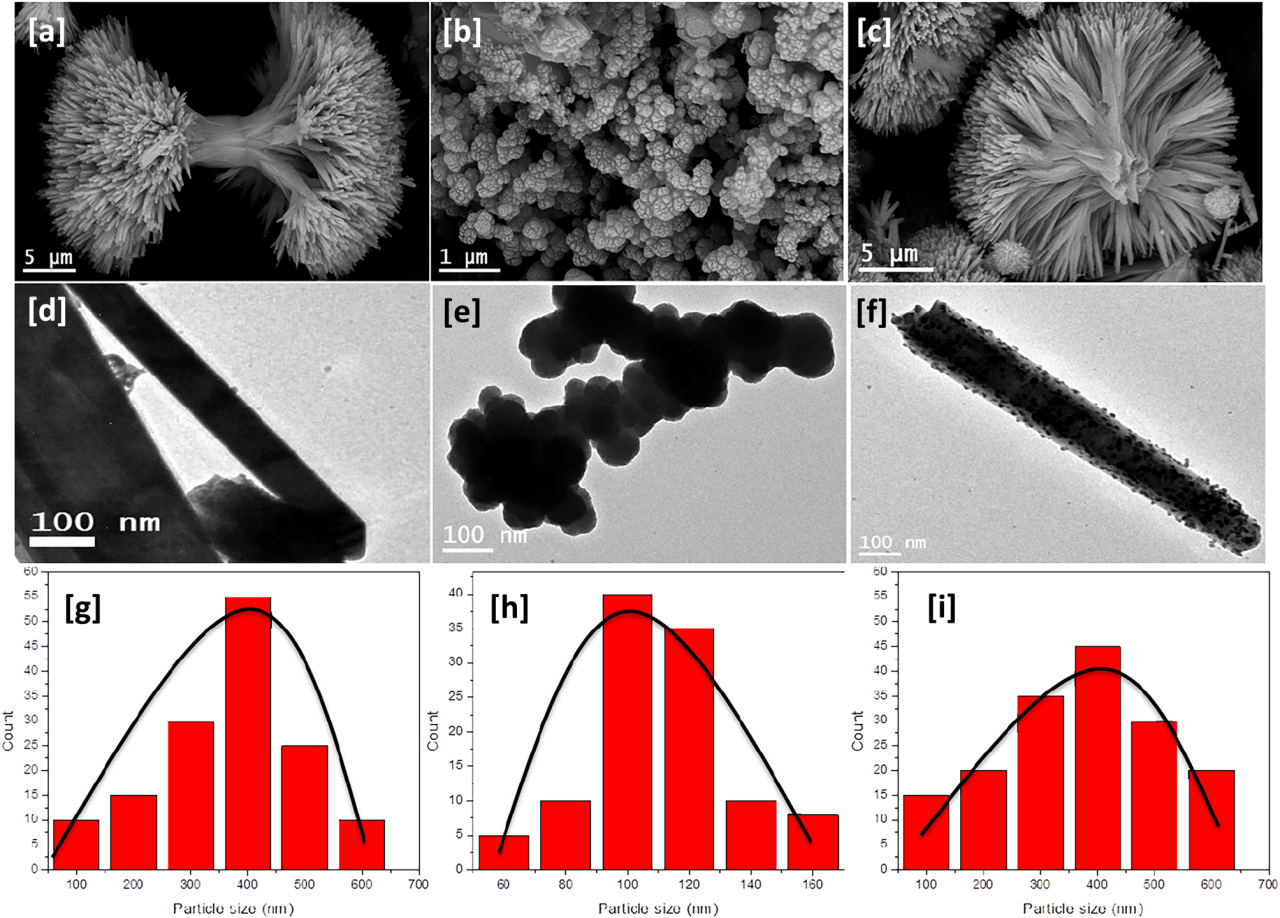
SEM of Ce-BTC (**a**), Mo(MnO_4_)_5_ (**b**), and Mo(MnO_4_)_5_@Ce-BTC (**c**), TEM of Ce-BTC (**d**), Mo(MnO_4_)_5_ (**e**), and Mo(MnO_4_)_5_@Ce-BTC (**f**), particle size distribution of Ce-BTC (**g**), Mo(MnO_4_)_5_ (**h**), and Mo(MnO_4_)_5_@Ce-BTC (**i**).

**Figure 4:**
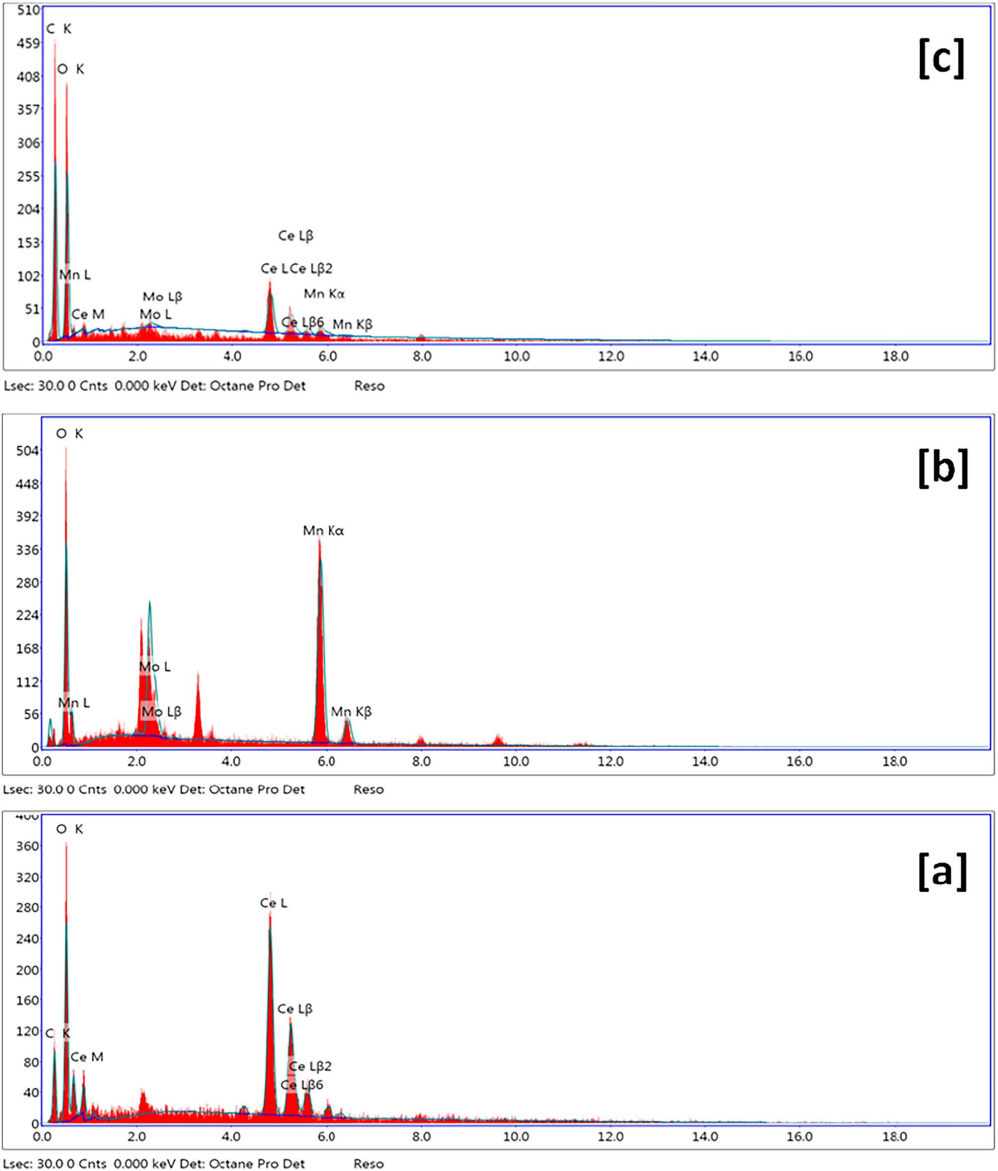
EDX of Ce-BTC (**a**), Mo(MnO_4_)_5_ (**b**), and Mo(MnO_4_)_5_@Ce-BTC (**c**).

Ce-BTC (Fig. 3a) has a morphology that is looks straw bundle-like with two fantails consisting of a bundle of outspread nanorods, which are closely bonded to each other in the middle, so we call it a “straw bundle-like”. The individual straw-sheaf has a length in the range of 5 μm and a middle diameter in the range of 2 μm. TEM image of a Ce-MOF crystals have rod-shaped particles that range in size from 2 to 6 µm (Fig. 3d) indicates that numerous nanorods with very high density are radially arranged from the center of the straw bundle structures. Furthermore, the chemical composition of the Ce-BTC was further investigated with EDX, which indicates that the architectures are made of Cerium, Carbon, and oxygen (Fig. 4a), confirming that these strawsheaves are formed from cerium and benzene-1,3,5-tricarboxylic acid. The FE-SEM image (Fig. 3b) showed that the Mo(MnO_4_)_5_ catalyst displayed spherical shaped particles, 1–2 μm in size, TEM image of a typical spherical shaped (Fig. 3e) indicates that numerous nanospher with very high density. Furthermore, the chemical composition of the Mo(MnO_4_)_5_ was further investigated with EDX, which indicates that the architectures are made of Mo, Mn, and O (Fig. 4b). The FE-SEM image of Mo(MnO_4_)_5_@Ce-BTC (Fig. 3c) displayed Urchin-like particles with 10 μm in size, it is clear that this procedure results in homogeneous distribution of the Mo(MnO_4_)_5_ nanoparticles across the Ce-MOF crystals; TEM image (Fig. 3f). Particle size distribution of Ce-BTC Mo(MnO_4_)_5_, and Mo(MnO_4_)_5_@Ce-BTC showed 400, 100, 450 nm in size, respectively (Fig. 3g–i). Furthermore, the chemical composition of the Mo(MnO_4_)_5_@Ce-BTC was further investigated with EDX, which indicates that the architectures are made of Mo, Mn, Ce and O (Fig. 4c).

**Figure 5:**
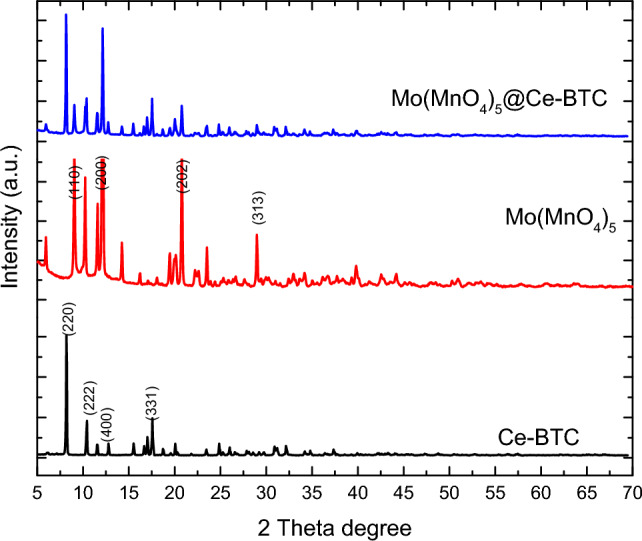
PXRD of Ce-BTC, Mo(MnO_4_)_5_ Mo(MnO_4_)_5_@Ce-BTC.

The XRD patterns of Ce-BTC, Mo(MnO_4_)_5_, and Mo(MnO_4_)_5_@Ce-BTC are shown in Fig. 5. The Ce-BTC XRD pattern was revealed by the present prepared compound, it is confirmed that the prepared Ce-BTC has a well-defined structure, with the major peak of 2*θ* appearing at 8.4°, 10.6°, and 18.1° revealed that the crystallinity of the Ce-BTC obtained was good and similar with that of the Ce-BTC in the earlier reports ^96^. The Mo(MnO_4_)_5_ XRD pattern was revealed by the present prepared compound, it is confirmed that the prepared Mo(MnO_4_)_5_ has a well-defined structure, with the major peak of 2*θ* appearing at 6.4°, 9.8°, 10.1°, 12.4°, 12.6°, 15°,22.1°, 25.2° and 28.2° revealed that the crystallinity of the Ce-BTC obtained was good. The Mo(MnO_4_)_5_@Ce-BTC XRD pattern was revealed by the present prepared compound, it is confirmed that the prepared Mo(MnO_4_)_5_@Ce-BTC has a well-defined structure, with the major peak of both Ce-BTC and Mo(MnO_4_) which revealed that the crystallinity of the Mo(MnO_4_)_5_@Ce-BTC obtained was good so, it has the same crystal structure. But there are a small shift and high intensity that points out the strong interaction between composite components. The XRD pattern indicates that the incorporation of Mo(MnO_4_)_5_ nanoparticles within the Ce-MOF crystals creates a supported catalyst without compromising the integrity of the Ce-MOF crystal structure.

**Figure 6:**
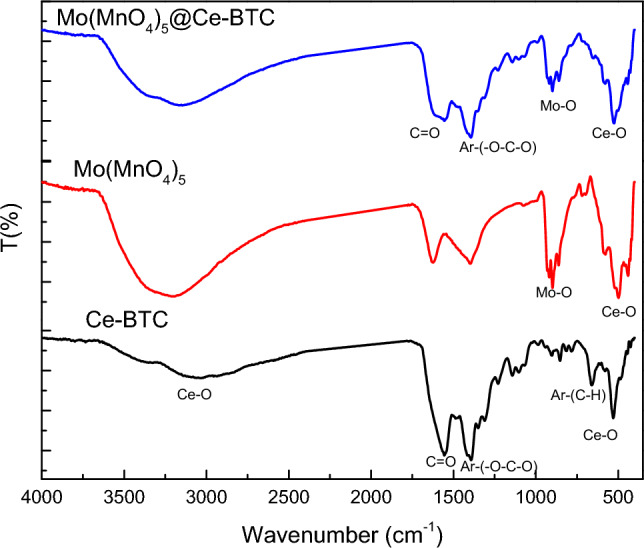
FTIR of Ce-BTC, Mo(MnO_4_)_5_, Mo(MnO_4_)_5_@Ce-BTC.

Figure 6 shows the Fourier transform infrared (FTIR) of all compound used in the present study, Ce-BTC, Mo(MnO_4_)_5_, Mo(MnO_4_)_5_@Ce-BTC. For prepared Ce-MOF crystals the spectrum shows the characteristic bands of the COO– groups of BTC^3−^ such as the 1612 cm^−1^ asymmetric vibration and the 1435 cm^−1^ and 1373 cm^−1^ symmetric vibration. In addition, low intensity bands of the Ce–O stretching vibrations are observed near 500–700 cm^−1^. The strong OH stretching band of water at 3450 cm^−1^ is attributed to physically adsorbed water molecules on the surface of the Ce-MOF crystals. These peaks all indicate that the Ce metal salt we have used has been coordinated with the 1,3,5-H_3_BTC ligand, and the Ce-MOF has been successfully prepared.

FTIR spectra of Mo(MnO_4_)_5_ showed absorption band around 850–1000 cm^−1^ corresponds to various Mo–O and Mo–O–Mo bonds. The bands at 400–750 cm^−1^ were related to stretching vibrations of O–Mn–O. The broad band at 3438 cm^−1^ is related to the adsorbed surface water molecule’s hydrogen-bonded O–H group. The absorptions around 1400 and 1641 cm^−1^ related to the vibration of Mo–OH and H–O–H bond deformation ^97^. The FTIR of the target Mo(MnO_4_)_5_@Ce-BTC showed the combination of the FTIR of both starting materials.

The optical properties of Ce-BTC and Mo(MnO_4_)_5_@Ce-BTC were investigated by UV–Vis diffuse reflectance spectroscopy (DRS) as illustrated in Fig. S1. The band gap energies (E_g_) of Ce-BTC was 2.68 eV, while Mo(MnO_4_)_5_@Ce-BTC showed the lowest band gap 2.65 eV, This low E_g_ value of Mo(MnO_4_)_5_@Ce-BTC catalyst indicated the highest visible light harvesting capability of the catalyst. Figure S2 present the Mott–Schottky curves of Ce-BTC and Mo(MnO_4_)_5_@Ce-BTC. The Mott–Schottky curves were measured to determine the band structures of Ce-BTC and Mo(MnO_4_)_5_@Ce-BTC. It can be seen from the figure that the Mott–Schottky curves of Ce-BTC and Mo(MnO_4_)_5_@Ce-BTC both had positive slopes, so Ce-BTC and Mo(MnO_4_)_5_@Ce-BTC were both n-type semiconductors.

BET analysis of Ce-BTC, Mo(MnO_4_)_5_, Mo(MnO_4_)_5_@Ce-BTC was investigated and represented in Table 1 in order to better understand the surface area and photocatalytic mechanism. The Mo(MnO_4_)_5_@Ce-BTC showed surface areas 484 m^2^/g and Ce-BTC showed 625 m^2^/g. The average pore volume of Mo(MnO_4_)_5_@Ce-BTC is 0.21 cm^3^/g, and Ce-BTC is 0.32 cm^3^/g. This confirm that Mo(MnO_4_)_5_ was incorporated with MOFs inside pores. Figure S3 shows the N_2_ adsorption–desorption isotherms of Ce-BTC and Mo(MnO_4_)_5_@Ce-BTC, it shows a type I isotherm confirming micropore character for the prepared materials.

**Table 1:** BET of Ce-BTC, Mo(MnO_4_)_5_, Mo(MnO_4_)_5_@Ce-BTC.

Sample	BET surface area (m^2^/g)	Pore volume (cm^3^/g)
Ce-BTC	625	0.32
Mo(MnO_4_)_5_	42	0.08
Mo(MnO_4_)_5_@Ce-BTC	484	0.21

### Photocatalytic urea production property

**Figure 7:**
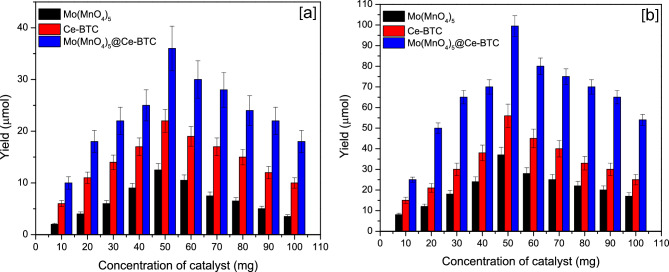
Effect of catalyst loading on the production of urea (**a**) dark conditions (**b**) light conditions.

Figure 7 showed the effect of catalyst loading (Ce-BTC, Mo(MnO_4_)_5_, Mo(MnO_4_)_5_@Ce-BTC) on the production of urea. For Mo(MnO_4_)_5_@Ce-BTC (Fig. 7a) showed higher activity than Mo(MnO_4_)_5_ and Ce-BTC. It showed 40 µmol/L at 50 mg catalyst in dark condition but in light condition it was reached 105 µmol/L. The catalyst dosage was increased from 10 to 50 mg (Fig. 7). When the catalyst loading was increased from 50 to 100 mg, the production yield was reduced due to the interaction with catalyst particles and prevented light from reacting with substrate molecules.

**Figure 8:**
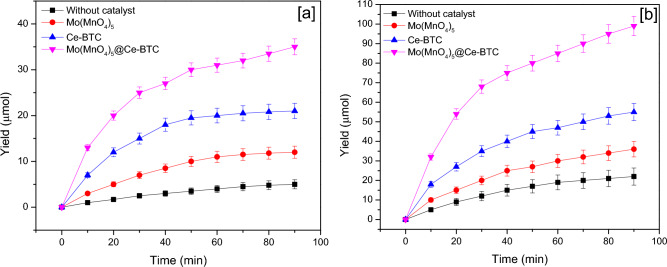
Effect of time on the production of urea (**a**) dark conditions (**b**) light conditions.

The as-synthesized Ce-BTC, Mo(MnO_4_)_5_, Mo(MnO_4_)_5_@Ce-BTC were applied as visible-light photocatalysts to produce urea. As shown in Fig. 8, the urea production was time-dependent. The yield of urea production increased with time until saturation. Mo(MnO_4_)_5_@Ce-BTC showed high production yield at 90 min with 105 µmol/L.

### Stability and recyclability of the catalyst

**Figure 9:**
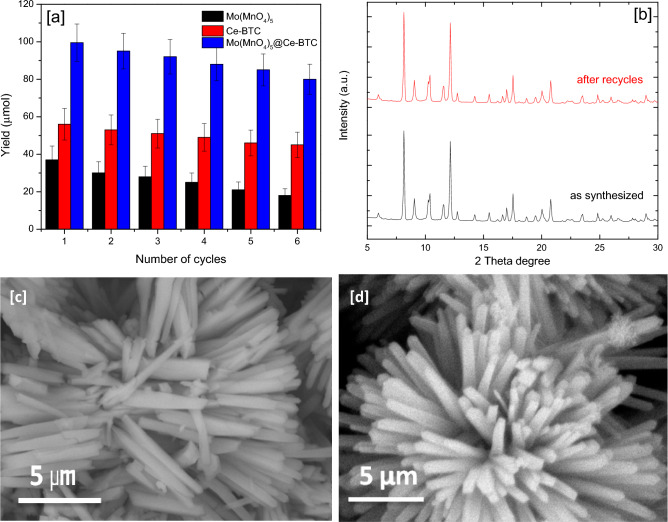
(**a**) Recyclability of Ce-BTC, Mo(MnO_4_)_5_, Mo(MnO_4_)_5_@Ce-BTC photocatalysts, (**b**) PXRD of Mo(MnO_4_)_5_@Ce-BTC before and after recycle, (**c**) SEM of Mo(MnO_4_)_5_@Ce-BTC after recycle, and (**d**) SEM of Mo(MnO_4_)_5_@Ce-BTC as synthesized.

One of the most important characteristics of a photocatalyst is that it must be stable, recyclable, and reusable after a few cycles. Experiments were carried out under ideal conditions to assess the stability and recyclability of the Ce-BTC, Mo(MnO_4_)_5_, Mo(MnO_4_)_5_@Ce-BTC photocatalyst. The catalysts were centrifuged after the reaction, washed three times with MeOH, and reused in the next reaction. This cycle was done six times in a row under similar conditions, and the yield of the product was determined. Figure 9a shows a summary of the findings.

The yield of urea generation only decreases by 20% after six repetitions. The XRD pattern of the recycled Mo(MnO_4_)_5_@Ce-BTC is comparable to that of the unused material (Fig. 9b), and the morphology of the material is not noticeably altered in the FE-SEM pictures (Fig. 9c), indicating that photocatalysis did not cause any structural damage to the Mo(MnO_4_)_5_@Ce-BTC(Fig. 9d). The leaching of active species (Mo^+5^, Mn^+2^ and Ce^+3^ ions) from the photocatalysts was determined using ICP-OES and the results showed that leached species remained below the detection limits of spectrophotometer.

### Suggested reaction mechanism of urea production

**Figure 10:**
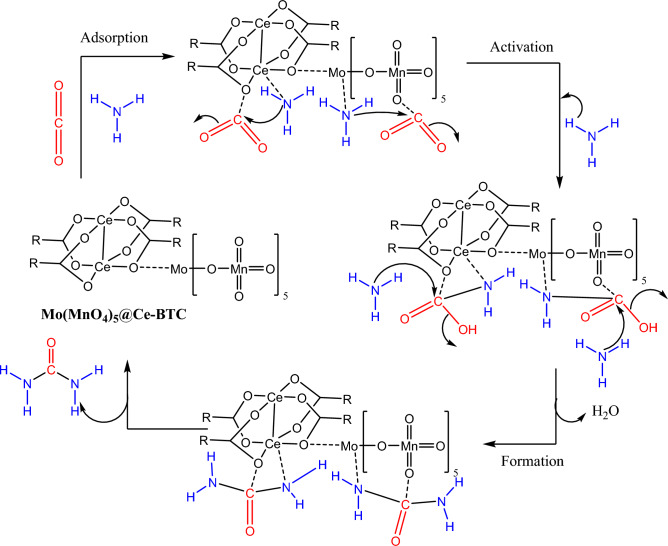
The suggested mechanism for the production of urea in presence of photocatalyst.

The photocatalytic coupling mechanism of CO_2_ and NH_3_ based on Mo(MnO_4_)_5_@Ce-BTC composite was suggested s shown in Fig. S4, the electrons migrated from the valence band (VB) to the conduction band (CB) and leave the holes in its VB. This is because of the difference in electrical potential, moreover, photogenerated electrons are migrated from the CB of the Ce-BTC to the CB of Mo(MnO_4_)_5_, in the same time, photogenerated holes will migrate from the VB of Ce-BTC to the VB of Mo(MnO_4_)_5_. This confirmed that generated electrons were the responsible for coupling mechanism. We suggest a method for production of urea by coupling of CO_2_ with NH_3_ (Fig. 10) depend on current information and previous works^18,98–100^. At first CO_2_ and NH_3_ molecules were adsorbed and connected with the metal center of the network and doped metal oxide nanoparticles. Therefore, it is speculated that the mechanism of photocatalytic generation of urea from CO_2_ and NH_3_ is that NH_3_ and adsorbed CO_2_ are coupled on Mo(MnO_4_)_5_@Ce-BTC , and the intermediate H_2_NCOOH is generated and after activation react with NH_3_ to form ammonium carbamate (H_2_NCO_2_NH_4_) which undergoses one-step hydrogenation, which generates urea through a multi-step process as shown reaction path :

$${\text{CO}}_{{2}} + {\text{ NH}}_{{3}} \to {\text{H}}_{{2}} {\text{NCOOH}}\quad \underrightarrow {{\text{NH}}_{3} } \, \quad {\text{H}}_{{2}} {\text{NCO}}_{{2}} {\text{NH}}_{{4}} \quad \underrightarrow { - {\text{H}}_{2} {\text{O}}}\quad {\text{H}}_{{2}} {\text{NCONH}}_{{2}}$$

In the presence of light, NH_3_ molecules participate in the addition reaction to connected CO_2_ and liberated water molecule. The final step is performing urea molecule and separate the photocatalyst.

## Conclusion

Ce-BTC and Mo(MnO_4_)_5_@Ce-BTC photocatalysts were synthesized and characterized using different techniques. The new composites worked as a photocatalysts for urea production, which leaded to the development of cleaner catalytic processes. The effects of different loading photocatalysts doses was investigated in order to find the best conditions for the production of urea; the results confirmed that 50 mg catalyst at room temperature produce high yield. The current Mo(MnO_4_)_5_@Ce-BTC photocatalysts, fortunately, may be regenerated and reused without losing activity, making it a viable alternative to homogenous basic reagents.

### Supplementary Information

Supplementary Figures.

## Data Availability

All data generated or analysed during this study are included in this published article.
