# Supplementary material for: Urea production via photocatalytic coupling of mixed gases (CO2/NH3) using Mo(MnO4)5 supported on Ce-BTC as nano-composite catalyst
Source: Sci Rep. 2024 Jul 6;14:15608. doi: 10.1038/s41598-024-65363-z (PMC11227513; doi:10.1038/s41598-024-65363-z)
Supplement: Supplementary file 1 — Supplementary Figures. [file 41598_2024_65363_MOESM1_ESM.docx]

**Supporting information**

Fig. S1.  Tauc plots of Ce-BTC and Mo(MnO_4_)_5_@Ce-BTC

Fig. S2 Mott–Schottky plots of Ce-BTC and Mo(MnO_4_)_5_@Ce-BTC

**Fig. S3.** Nitrogen adsorption/desorption isotherms


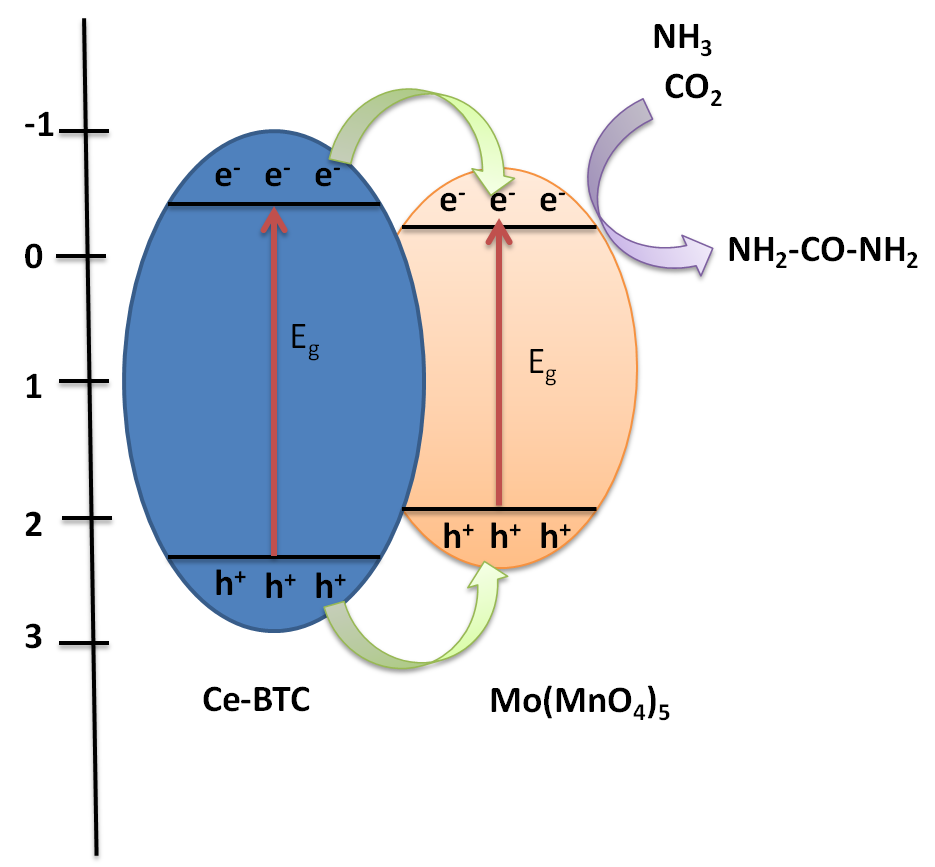


**Fig. S4.** Schematic of the separation and transfer of photo-generated charge carriers in Mo(MnO_4_)_5_@Ce-BTC
